# Availability and use of assistive technologies at selected South African public libraries

**DOI:** 10.4102/ajod.v12i0.1141

**Published:** 2023-08-31

**Authors:** Takalani M.M. Mamafha, Patrick Ngulube, Luyanda Dube, Sindile A. Ngubane

**Affiliations:** 1 Department of Information Science, College of Human Sciences, University of South Africa, Tshwane, South Africa; 2 Department of Interdisciplinary Research and Postgraduate Studies, College of Graduate Studies, University of South Africa, Tshwane, South Africa; 3 Institute for Open and Distance Learning (ODL), College of Education, University of South Africa, Tshwane, South Africa

**Keywords:** assistive technologies, persons with visual impairment, print disability, City of Ekurhuleni (CoE), City of Johannesburg (CoJ), South Africa

## Abstract

**Background:**

Assistive technologies (ATs) enable persons with visual impairment (PwVI) to equitably benefit from public library resources and services as their sighted counterparts. However, the extent to which this facility is available and used at public libraries in less-developed countries remains largely unknown.

**Objectives:**

This study reports on the investigation done on the availability and use of ATs by PwVI at public libraries in the cities of Ekurhuleni and Johannesburg in South Africa.

**Method:**

The study used a multimethod and explanatory sequential design in which data were collected through questionnaires administered with 131 librarians and interviews held with 10 PwVI.

**Results:**

The findings of the study point towards inadequate availability of computers with internet services, audiotapes, screen magnifying and reading software, and these were used for, among others, leisure, research, job searching and communication.

**Conclusion:**

The study concludes that certain ATs were inadequately available and used by PwVI at some libraries in the cities of Ekurhuleni and Johannesburg. The study recommends training for PwVI on how to use ATs, marketing of available ATs, training of staff on how to render AT-based services as well as management availing adequate budget for the development of AT-based collection.

**Contribution:**

The study contributes to the understanding of the types of ATs available and used by PwVI in public libraries.

## Introduction

The availability and use of assistive technologies (ATs) by persons with visual impairment (PwVI) in public libraries are topical and part of the global inclusive agenda towards the implementation of the Sustainable Development Goals (SDG) and the United Nations Convention on the Rights of Persons with Disabilities (UNCRPD) (Tebbutt et al. 2016). Given their role as equalisers of information access, ATs in public libraries have radically transformed service delivery to PwVI. Notably, these have been denied opportunities to access and use library resources and services due to decades of institutionalised global discrimination (Majinge & Stilwell 2013). Prior to introducing ATs in public libraries, PwVI had limited access to and use of electronically available library resources and services (Lee 2005) as they largely relied on library staff for assistance. However, PwVI presently enjoy freedom of independence and choice in public library service due to the adoption and introduction of applications such as e-books, electronic mail and the internet (Lee 2005). These devices are essential tools in restoring PwVI’s dignity and self-esteem in public library usage (Lee 2005).

The World Health Organization (WHO) defines assertive technology is any item, software programme, equipment or product system that is used to maintain, increase or improve the functional abilities of persons with disabilities (WHO 2018). In the context of libraries, ATs refer to hardware and software devices that assist persons with disabilities to access computers or other information technologies. For PwVI library users, the combined hardware and software include: (1) reading machine, (2) video conferencing, (3) computers and their internet-based services, (4) screen reader, (5) braille writing equipment, (6) braille translation software, (7) braille embosser, (8) scanners and (9) closed-circuit television (CCTV) that enable PwVI to access information resources and services such as electronically accessible books and newspapers (Adetoro 2011; Anis 2017; New York Public Library 2018). Besides compensating for the vision impairment and facilitating access to information and communication of the PwVI, these devices, according to Eligi and Mwantimwa (2017), provide an opportunity for users to learn new skills required for them to benefit from electronically based services same way as their sighted counterparts. For instance, ATs enable PwVI ‘who are partially sighted persons who may not read standard print with remedial lenses’ (WHO 2018) to chat with friends, study, conduct research and read from original information sources available on the World Wide Web (WWW) at the same time as everyone else (Golub n.d.).

Public libraries in developed countries of the world such as the United Kingdom, the United States, Canada and Australia are adopting and using ATs as a way of complying with their countries’ disability laws (Kinnell, Yu & Creaser 2000). For instance, there is the *Disability Discrimination Act* (DDA) in the United Kingdom (UK) and the *Americans with Disabilities Act* (ADA) in the United States. In the case of the Global South, South Africa has developed numerous policies and legislations that advocate for speedy and effective implementation and use of ATs at public libraries with a view to ensuring equalisation of access by all to library services. These include the Library and Information Services (LIS) Transformation Charter, National Policy for Libraries and Information Services in South Africa, South African Public Library and Information Services Bill 2012, as well as *Batho Pele* (People First) White Paper on Transforming Public Service Delivery, which focuses on giving improved customer service to users of government service’s (Department of Arts and Culture 2018; Department of Social Development n.d; Gauteng Provincial Government n.d; Mamafha, Ngulube & Ndwandwe 2016; National Library of South Africa [NLSA] 2018). Developments in these principles, policies and legislations were influenced by the government’s realisation that ATs have the potential to provide hope in overcoming access barriers which PwVI have been subjected to for several decades (Mamafha et al 2016).

Cities of Ekurhuleni (CoE) and Johannesburg (CoJ) Municipality libraries, which are the subjects of this study, are, therefore, among libraries in South Africa that should have certain types of ATs to enable PwVI to equally access and use library resources and services as their sighted counterparts. This is in line with the International Federation of Library Associations and Institutions’ Manifesto for Libraries Serving Persons with a Print Disability, which aims to improve and promote accessible LIS to PwVI. The availability of ATs in these libraries would ensure equalisation of access to computerised resources and services for all irrespective of a person’s age, gender or nature of the disability.

### Context of the study

Public libraries in the CoE and CoJ municipalities are located in the Gauteng Province of South Africa. There are 47 libraries in CoE located in the city’s three regions, which are South, North and East (City of Ekurhuleni 2021). The CoJ has 86 libraries that are situated in its seven regions that are A–G (Joburg 2018). The selection of libraries for the study was based on their self-proclaimed status of being leaders in service delivery to communities with well-developed telecommunication infrastructure. Given this proclamation, it was necessary to establish whether targeted libraries had relevant devices and services for PwVI. Among the services rendered by these libraries include, but are not limited to, lending, reference, interlibrary loan, computer and internet-based services, fax, printing and photocopy, scanning, lamination and wireless network (Joburg 2018; Mamafha et al 2016). Public libraries in the two cities have transformed into centres of learning, allowing the public to have access to a range of information sources such as online magazines, audio material and electronic books (Ekurhuleni Metropolitan Municipality [EMM] n.d.; Joburg 2018). Furthermore, e-learning classrooms equipped with computers, giving users access to online courses such as coding, as well as access to e-resources including online academic sites, online newspapers and articles, are also available in some libraries (Joburg 2018; Mamafha et al 2016). Despite these positive developments at libraries in these cities, questions abound about whether these advancements extend to the availability of special access devices for PwVI against decades of global service inaccessibility for persons with disabilities. Evidently, library services have been designed to suit the needs of the sighted more than PwVI (Ibenne 2012; Majinge & Stilwell 2013), hence this study.

Against the above background, this study aimed at establishing whether ATs were available and readily in use at public libraries in CoE and CoJ. This was necessary to support the demands for libraries’ continued investment on ATs and their related services where they were available and ensure their effective utilisation. Alternatively, it sought to motivate public libraries’ acquisition of ATs where they were unavailable to facilitate improved access to services for PwVI. In the process of establishing ATs’ availability and use, reasons for their use and nonuse were also looked at. Furthermore, the difficulties faced by users in using ATs and how they could be alleviated were also investigated. Although a similar study conducted by Kinnell et al. (2000) found that certain ATs were available and used at Manchester city libraries, it was dated and based on the situation within the context of UK public libraries at the time. This study is aimed at establishing baseline understanding of the phenomenon within the context of public libraries in South Africa given the scarcity of research on the subject. The study was guided by the following research questions that were informed by the International Federation of Library Associations and Institutions (IFLA) manifesto mentioned earlier:

- What types of ATs are available and used by PwVI at the cities of Ekurhuleni and Johannesburg libraries?
- What are the reasons for the use of ATs by PwVI at the cities of Ekurhuleni and Johannesburg libraries?
- What difficulties are faced by PwVI in using ATs at public libraries in the cities of Ekurhuleni and Johannesburg?
- How could the difficulties faced by PwVI in using ATs at Ekurhuleni and Johannesburg public libraries be alleviated?

### Conceptual framework

The IFLA Manifesto for Libraries Serving Persons with a Print Disability provided a conceptual framework of the study to better understand the types of devices that public libraries in Ekurhuleni and Johannesburg should have to improve access and use of library resources and services by PwVI, thus enabling service inclusivity. The manifesto recommends that for libraries to meet the identified needs of users with a print disability, they should: (1) have as part of their core services, certain technological devices, equipment and facilities that comply with universal design principles to enable persons with a print disability to access and use the available technologies, (2) improve the information and communication technologies (ICTs) skills of PwVI through training, (3) partner with relevant stakeholders in the service and (4) involve persons with a print disability, including organisation or groups representing them, in the planning, development and ongoing delivery of services (IFLA 2012). The first recommendation resonates well with this study, thus implying that for the PwVI to have equal access to all public libraries’ information and services, public libraries should provide specialised access devices that comply with universal design principles. These, according to the checklist developed by Irvall and Nielsen (2005) for IFLA, include accessible websites, audio tapes, compact disc (CD), digital video disc (DVD) or digital accessible information system (DAISY) players. Although evidence of the use of this manifesto by other scholars in previous studies could not be found, which brings another important dimension of originality to this study, it provided a foundation for the current research and structure within which a problem under analysis could be understood while acting as a footprint for future researchers. This framework helped in formulating the research questions as well as explaining the relationship between public libraries and their users in the context of ATs availability and use.

## Literature review

### Types of assistive technologies available for persons with visual impairment in public libraries

Adetoro (2011) opines, ‘the principle underpinning library and information service provision to persons with visual impairment should be that of availability and equality of access to information materials’. Literature on the types of ATs available for PwVI in public libraries is limited, generally dated and within the context of libraries in developed countries. Kinnell et al. (2000), for instance, conducted a similar study in the UK whose results suggested that reading aids, including magnification aids (CCTV, zoom-text computer screen magnification software), speech recording facilities (those linked to reading machines to permanently record the spoken text), speech systems (reading machine and various screen readers) and alternative output facilities (embossers that produce disk files in Braille) were available at Manchester City libraries. The same study found that reading machines, printers and other related aids were available at Tameside libraries. A study by Share the Vision (1991) revealed that at least one CCTV was available at 38% of selected UK public library authorities, while at least one reading machine was available at 23% of library authorities. Furthermore, the study found that 7% of library authorities had computerised Braille input or output devices while 26 out of 61 libraries had reading aids including magnifiers, cassette players, reading machines, CCTV and even computerised devices and software. Also, the study established that nine authorities had only magnifiers and/or cassette players. The literature review also reveals that the Heiskell Braille and Talking Book Library in New York had handheld magnifiers, braille writer, a Perkins Smart Brailler, an inTact sketchpad for creating tactile drawings, a Victor Reader and Apple computer (New York Public Library 2018). In Nigeria, Adetoro (2014) conducted a study on information provided to the visually impaired and found that the availability of alternative formats was inadequate while electronic resources were unavailable in public libraries. In South Africa, the extent of availability of public library ATs for PwVI is unknown. This is despite the claim made by the South African Library for the Blind (SALB) (n.d.), Rowland (2008) and Education News South Africa (n.d.) that, advanced reading assistive devices including document reader, Victor reader for audiobooks, CDs and DVDs are available in South African public libraries in the form of a mini-library service sponsored by the SALB. This claim has yet to be empirically validated, hence this study.

### Use of assistive technologies by persons with visual impairment in public libraries

Though the literature on the subject is dated, there is consensus among researchers regarding the types of ATs used by the PwVI including reasons for their usage. Bruce, McKennel and Walker in Adetoro (2011) found that PwVI users of UK libraries used personal readers, tape recording and Braille readers. Davies, Wisdom and Creaser in Lewis (2004) found that a majority of the 581 PwVI using UK public libraries mostly used speech software, text enlarger and a screen magnifier. Lewis (2004) also found that electronic reference devices including CD-ROM and WWW were used by PwVI at the Royal National Institute for the Blind (RNIB) public library. This was so even though very few (12.5%) of the sampled population used the devices or service compared to 87.5% of those who did not use it. The use of an electronic library catalogue was also acknowledged by the same study. In India, the study in the leading National Capital Region (NCR) libraries by Kumar and Sanaman (2013) found that audiobooks on CDs, DVDs and DAISY books were the most used electronic devices and resources in the libraries. However, Pillai (2013) found that some users of libraries in India did not use electronic access devices and resources due to perceived complexity and difficulties in using them. In Africa, the extent of use of public libraries’ ATs for PwVI is generally unknown. Although the study by Adetoro (2014) found that the use of information material in an alternative format in Oyo, Ekiti, Osun and Ondo in Nigeria was limited by availability, there is generally a lack of empirical evidence on the extent to which PwVI use ATs within the context of other African public libraries including South Africa. In fact, a similar study by Mamafha et al. (2016) in South Africa mainly focused on the use of technologies for the mainstream users of Ekurhuleni libraries where it was found that the facility was highly utilised by most users.

Common reasons for the use of ATs by PwVI at public libraries included information search, general browsing, online shopping, job searching, studying towards a qualification, chatting, news, banking, entertainment and instant messaging (Lee 2005). For instance, Wallis, Morley and Wallis (2000) found that users of the Royal Victorian Institute for the Blind Library and Information Services (RVIBLIS) in Australia used voice print devices to access the library catalogue, listen to the latest titles available and listen to a catalogue listing of any title, including a brief annotation. The technology, according to the study, was also used for ordering books and accessing the full editorial text of publications such as *The Age, Australian Financial Review, Business Review Weekly, Sydney Morning Herald, The Melbourne Herald Sun, The Adelaide Advertiser* and *The Australian* (Wallis, Morley & Wallis 2000).

### Difficulties faced by persons with visual impairment in using assistive technologies in public libraries

The PwVI may, in fact, face a lot of difficulties in using ATs in public libraries. The setback is likely to cut across all the diverse types of libraries, namely, school, academic or public. Such impediments arise because of the lack of relevant equipment necessary for public libraries to respond to the ever-increasing reading and information needs of the users (Adetoro 2009). Furthermore, some PwVI lack the knowledge and skills to use their tailor-made ATs in public libraries. Adetoro (2009), Rayini (2017) and Eligi and Mwantimwa (2017) identify limited financial and human resources, staff attitudes, technophobia and the lack of appropriate disability-friendly policies and their proper implementation. For instance, the study by the Library of Congress (LOC) (2012) in the United States found that the major difficulty faced in providing library services to PwVI through the National Library Service (NLS) was PwVI’s (and their next of kin) lack of knowledge to operate the technology due to skill deficit. Ejedafiru and Oghenetega (2010) and the National Library of South Africa (2014) identified limited financial and human resources as some of the difficulties faced by PwVI in Nigeria and South Africa, respectively.

Although the availability and use of ATs by PwVI in academic libraries have been subjects of numerous investigations in Nigeria, Tanzania and other developing countries in recent years (Abdelrahman 2016; Eligi & Mwantimwa 2017; Ibenne 2012; Kumar & Sanaman 2013; Obichere 2011; Onoyeyan 2019), this subject matter remains largely unexplored within the context of public libraries, particularly in South Africa. The ensuing gap has created scope for this study.

## Research methods and design

This article adopted the pragmatist paradigm using explanatory sequential design with a view of looking at the studied phenomenon from a broader perspective (Creswell 2014:10). Multi-methods were used for the researcher to get comprehensive viewpoints from study participants on the PwVI’s use of ATs at selected public libraries. The integration of quantitative and qualitative methods occurred at the research questions, data collection, results and discussion stages of the research. The use of multi-methods enabled the researcher to collect statistical data from librarians using questionnaires that yielded information on the types of ATs available and used at selected libraries. It also allowed for the collection of descriptive data from PwVI using an interview schedule that provided insight into why ATs and their related services were used. The population of the study was all 131 CoE and CoJ public libraries combined, of which 45 belonged to CoE and 86 belonged to CoJ. Lists of libraries in the two cities were provided by the librarians and validated by their respective websites after permission for the study to be conducted was granted by the respective directors. Given its manageable size, the entire population of this study was sampled using total population sampling. Within the libraries, all 131 librarians in charge of the selected libraries were targeted as respondents in the initial phase of the study. Also targeted for the study were the entire 10 registered PwVI (five from CoE and five from CoJ libraries).

Triangulation of data collection instruments was adopted using e-mailed questionnaires and interviews to gather data from librarians and PwVI, respectively. This was to ensure the reliability and validity (quantitative) and trustworthiness (qualitative) of the data gathered. Data collection happened sequentially in which data (both in quantitative and qualitative form) were collected first using questionnaires. This was followed by the gathering of qualitative data from PwVI by means of an interview schedule. Prior to data collection, permission to conduct the study was sought from and granted by the LIS directors of the two cities. Meanwhile, an ethical clearance certificate was obtained from the University of South Africa (UNISA). Questionnaires consisted of eight mainly quantitative questions in which librarians had to select the most appropriate answers from several given alternatives. However, librarians were also asked to provide answers to qualitative-oriented questions on the difficulties faced by PwVI in using ATs and how these could be alleviated. Librarians were given 3 weeks to complete and return questions, which was to reduce errors that would have occurred had limited time been given for their return. The research questions wherein the questionnaire was applied as a data collection tool were the following: (1) what types of ATs are available for PwVI at Ekurhuleni and Johannesburg libraries? (2) what are the reasons for the use of ATs by PwVI at Ekurhuleni and Johannesburg libraries? (3) what difficulties are faced by the PwVI in using ATs at Ekurhuleni and Johannesburg libraries and (4) how could they be alleviated?

Meanwhile, telephone interview sessions (which each lasted for 15 minutes in which respondents answered six questions) were held with 10 targeted PwVI to gather more detailed information on their use of ATs as well as difficulties encountered during usage. A digital audio voice recorder was used in recording the interviews by converting the recorded speech to a text transcript. The research questions applicable to the interview schedule as a data collection instrument were the following: (1) what are the reasons for the use of ATs by PwVI at Ekurhuleni and Johannesburg libraries? and (2) what difficulties are faced by PwVI in using ATs at Ekurhuleni and Johannesburg libraries, and how could they be alleviated? Consent for the study was sought from participants who were all informed that their participation was voluntary and that they could withdraw from the study at any given time. Meanwhile a voice recorder was used to record each of the interviews with 10 PwVI. Ten librarians and four PwVI portraying the actual participants in the final study were carefully chosen for the pretest. The total number of questionnaires returned for the main study was 80 (30 from CoE and 50 from CoJ), representing 61% of the questionnaires received and analysed. Quantitative data were presented in tables and analysed by showing central tendency and spread. Qualitative data were analysed through verbatim transcription, which is converting audio recordings of interviews to text format. In this instance, the recorded responses were converted into text and meaning was drawn from the data collected.

### Ethical considerations

An application for full ethical approval was made to the UNISA Department of Information Science Research Ethics Review Committee, and ethics consent was received on 01 December 2017. The ethics approval number is ‘2017_TMMMamafha_34666249_001’.

Informed consent was obtained from all individual participants involved in the study. These include written consent from librarians and oral consent from the PwVI. The preference for oral consent with the PwVI was informed by its usability when dealing with persons who may not read or understand the standard text unless it is converted into the language they are acquainted with. Also, researchers were unable to find a knowledgeable and willing participant to convert the standard text to braille, which could be read by PwVI, at a reasonable cost at the time of the study. The measures taken to maintain the confidentiality of the data was as follows:

- The participants’ personal information was not required for the purpose of this study as a way of guaranteeing their anonymity.
- Information provided by participants was not used for anything other than the purpose of the study.
- Information provided by participants was not to be used to disadvantage them in any way soon.

## Results

The results that are presented in this section are based on the research questions of the study.

### Age distribution of respondents

The profiles of librarians reveal that the majority were older than 40 years. These were followed by those between the ages of 35 and 40 years. Those between the ages of 26 and 34 years were few as seen in Table 1. In South Africa, middle and senior library management positions are occupied by older persons who will be eligible for retirement in few years to come (Van der Walt & Du Plessis 2010).

**TABLE 1:** Age profiles of librarians.

Variables	City of Ekurhuleni	City of Johannesburg	Total frequency	Total percentage of respondents
**Age (years)**				
Between 26 and34	4	4	8	10
Between 35 and 40	12	10	22	29
Above 40	14	36	50	61

**Total**	**30**	**50**	**80**	**100**

The above profiles for PwVI show that the majority were above the age of 40 years except for one who was within the age range between 36 and 40 years. There were more males in this category than females as seen in Table 2 Meanwhile, the occupation of the PwVI reveals that the majority were unemployed while the remaining few were university students as shown in Table 2. In South Africa, a report by Blind South Africa (BLINDSA) in the *Daily Maverick* (2022) indicates that over 90% of blind and visually impaired people are unemployed.

**TABLE 2:** Age profiles of persons with visual impairment.

Variables	City of Ekurhuleni	City of Johannesburg	Total frequency
**Age (years)**			
Between 18 and 35	0	0	0
Between 36 and 40	1	0	1
Above 40	4	5	9
**Gender**			
Male	4	3	7
Female	1	2	3
**Occupation**			
Student	2	1	3
Employed	0	0	0
Unemployed	3	4	7

### Types of assistive technologies available for persons with visual impairment at Ekurhuleni and Johannesburg public libraries

The first objective of this study was to assess the availability of ATs at public libraries in Ekurhuleni and Johannesburg for PwVI. At first, librarians were asked to answer, in a yes or no question format, whether ATs were available in their libraries. In response, 18 (22%) librarians indicated ‘yes’ against 62 (78%) who stated that they were not. In terms of municipalities, 14 (18%) CoE librarians indicated that AT-based service was available against 16 (20%) who said it was unavailable. A similar trend was noted in CoJ, wherein only 4 (5%) librarians indicated that their libraries had ATs compared to 46 (57%) that said theirs did not have them. Further details of the types of ATs available in 18 (22%) of the targeted libraries were sought from librarians of which 14 (18%) were from CoE while 4 (5%) were from CoJ. The finding revealed that audiotapes (CDs, DVDs and DAISY players), computers with internet services, scanners, screen magnifiers, screen readers and braille displayers were inadequately available at some of the targeted libraries as shown in Table 3.

**TABLE 3:** Types of assistive technologies available for persons with visual impairment at public libraries in Ekurhuleni and Johannesburg (*N* = 18).

Types of ICTs available	Ekurhuleni municipality	Johannesburg municipality	Total frequency
Braille displayer	1	-	1
Braille translator	-	-	-
Close-circuit television (CCTV)	-	-	-
Scanner	4	1	5
Screen reader	2	-	2
Screen magnifier	2	-	2
Computer with internet services	9	1	10
DAISY player	11	-	11
Fax machine	4	2	6
Touch Screen	-	-	-
CDs	12	4	16
DVDs	12	3	15

ICT, information and communication technologies; DVD, digital video disc; DAISY, digital accessible information system; CD, compact disc.

It is clear from Table 3 that CDs and DVDs were equally available in most libraries in CoE. Also available were daisy players and computers with internet services in the municipality, although in a limited capacity. Furthermore, fewer libraries had fax machines, scanners, screen readers, screen magnifiers and braille displayers as shown in Table 3. In CoJ, CD, DVDs, fax machines, computers with internet and scanners were available at few libraries as shown in Table 3. In Nigeria, Adetoro (2014) found that the availability of alternative formats was limited while electronic resources were unavailable.

### Use of assistive technologies at Ekurhuleni and Johannesburg public libraries by persons with visual impairment

The second objective of the study was to determine the use of ATs at public libraries in Ekurhuleni and Johannesburg by PwVI. Eighteen librarians whose libraries had ATs above were asked (in a yes or no question format) to indicate whether the available ATs were used or not. In response, eight (10%) librarians, of which six (8%) were from CoE and two (3%) were from COJ, indicated that ATs in their libraries were used by PwVI, while 10 (13%) librarians, of which 8 (10%) were from CoE and 2 (3%) from COJ, stated that theirs were not utilised. This finding is consistent with that of the subsequent qualitative study, which established that most PwVI (6 out of 10 of which 3 were from CoE and 3 from CoJ) did not use ATs.

Meanwhile, respondents whose library ATs were used were to identify the types of ATs used and their responses are provided in Table 4.

**TABLE 4:** Types of assistive technologies used by persons with visual impairment at public libraries in Ekurhuleni and Johannesburg (*N* = 8).

Assistive technologies used	Ekurhuleni municipality		Johannesburg municipality		Total Frequency	Total percentage of respondents
	*n*	%	*n*	%		
Braille displayer	-	-	-	-	-	-
Braille translator	-	-	-	-	-	-
Close-circuit television	-	-	-	-	-	-
Scanner	-	-	-	-	-	-
Screen reader	-	-	-	-	-	-
Screen magnifier	-	-	-	-	-	-
Computer with internet services and disc operating system (DOS)	1	1	1	1	2	3
DAISY	2	3	-	-	2	3
Fax	-	-	-	-	-	-
Touch Screen	-	-	-	-	-	-
CDs	2	3	2	3	4	5
DVDs	2	3	1	1	3	4

DVD, digital versatile disc; CD, compact disc; DAISY, digital accessible information System.

Table 4 clearly reveals that a range of devices including CDs, DVDs, DAISY players and computers with internet services were used in CoE libraries but on a limited scale. The situation is almost the same in CoJ libraries where CDs, DVDs and computers with internet services were used by PwVI. This finding is consistent with that of the subsequent qualitative strand, which revealed that PwVI used audiotapes (CDs, DVDs and DAISY players) and computers with internet services as shown in the verbatim responses below:

‘I use CDs in the library to listen to music as well as to read books loaded in those CDs.’ (46-year-old, female, CoE library user)

‘I use audio CDs, tape audio and victor reader [*victor recorder*] to listen to the story and whatever they come with.’ (60-year-old, female, CoJ library user)

‘I use a computer with internet to browse through the web for information.’ (40-year-old, male, CoE library user)

‘I use computers with internet for the purpose of conducting some research for my university study.’ (56-year-old, male, CoJ library user)

The study in the leading NCR libraries by Kumar and Sanaman (2013) found that audio books on CDs, DVDs and DAISY books were India’s most used AT devices.

Furthermore, the above eight librarians who stated that ATs in their libraries were used were asked to provide, choosing from the list provided, what they considered to be the reasons for PwVI’s use of ATs. Among the reasons provided by CoE librarians included study, leisure or general browsing, independent learning and job searching. The reasons for ATs usage in CoJ libraries followed almost a similar trend in that independent learning, job searching, banking, access to the internet and study purpose were identified as reasons for their usage as shown in Table 5.

**TABLE 5:** Reasons for the use of assistive technologies by persons with visual impairment at public libraries in Ekurhuleni and Johannesburg (*N* = 8).

Reasons for use of ATs	Ekurhuleni municipality	Johannesburg municipality	Total frequency
For study purpose	4	1	5
Independent learning and/or research	2	1	3
Leisure or general browsing	5	2	7
Access to internet	-	1	1
Online shopping	-	-	-
Job searching	1	1	2
Banking	-	1	1
Communicate through email	-	-	-
Downloading books from BARD	-	-	-
Downloading books from JAWS	-	-	-
Downloading books from screen reader	-	-	-
Other (specify)	-	-	-

AT, assistive technologies; BARD, braille and audio reading download; JAWS, job access with speech.

Consistent with the aforementioned finding, the subsequent qualitative result revealed that PwVI used ATs for leisure, study, research and web browsing as reflected in the following responses:

‘Listening to music and reading books loaded to the CDs is the main reason for my use of library ATs.’ (46-year-old, female, CoE library user)

‘I use Internet in the library to get information for research purpose.’ (56-year-old, male, CoJ library user)

‘Browsing the web in order to access information is the main reason I use library ATs.’ (40-year-old, male, CoE library user)

The study by Lee (2005) also found that information search, general browsing, online shopping, job searching, studying towards a qualification, chatting, news, banking, entertainment and instant messaging were some of the reasons for PwVI’s use of ATs in libraries.

Meanwhile, 10 (13%) librarians, of which 8 (10%) were from CoE and 2 (3%) were from CoJ, whose library ATs were not used requested to provide (by selecting from the list of given possible answers) what they considered to be the reasons behind the non-use of the ATs by PwVI. Responses are provided in Table 6.

**TABLE 6:** Reasons for nonuse of assistive technologies by persons with visual impairment at Ekurhuleni and Johannesburg public libraries (*N* = 10).

Reasons for nonuse of ATs	Ekurhuleni municipality		Johannesburg municipality		Total frequency	Total percentage
	*n*	%	*n*	%		
Unavailability of preferred ATs in the libraries	6	7	2	3	8	10
Perceived complexity of ATs	6	7	-	-	6	7
PwVI’s lack of skills to use the technologies	3	4	-	-	3	4
PwVI’s lack of knowledge of the existence of ATs in public libraries	6	7	-	-	6	7
Other (specify)	-	-	-	-	-	-

PwVI, persons with visual impairment; AT, assistive technologies.

Table 6 shows that the unavailability of preferred ATs in libraries was regarded as the reason for the nonuse of the technologies in both the study settings. Nevertheless, some librarians from CoE viewed the perceived complexity of ATs as the reason behind their nonusage while others from the same municipality thought PwVI’s lack of knowledge of the existence of ATs in public libraries was the reason behind nonutilisation of ATs. Few librarians from CoE selected PwVI’s lack of skills to use the technologies as the reason.

The ensuing qualitative study yielded similar results in that unavailability of ATs, difficulties associated with the use of ATs, users’ lack of ATs usage skills and lack of knowledge of the existence of ATs in libraries were identified by PwVI as reasons for nonuse of the technologies at libraries in the two cities as reflected in the following responses:

‘I prefer to use the computer with JAWS software to download books, but the facility is not available in the library I am registered with, hence my none-use of other available equipment.’ (55-year-old, male, CoE library user)

‘I do not have the skills necessary to use ATs available, hence my reliance on staff, whom themselves do not seem to have the skills required to use the facility.’ (62-year-old, female, CoJ library user)

Librarians were further requested to indicate whether they considered ATs in public libraries to be ‘very useful’, ‘less useful’ or ‘not useful’ in fast-tracking service delivery to the PwVI. While all respondents answered this question, most respondents from both CoE and CoJ were of the view that ATs were ‘very useful’. However, some believed that ATs were less useful while the least number of respondents from both cities were of the view that ATs were not useful as shown in Table 7.

**TABLE 7:** Usefulness of assistive technologies to persons with visual impairment in selected public libraries (*N* = 80).

ATs usefulness	City of Ekurhuleni		City of Johannesburg		Total respondents	%
	*N*	%	*n*	%		
Very useful	24	30	38	48	62	78
Less useful	3	4	8	9	11	13
Not useful	3	4	4	5	7	9

**Total**	**30**	**38**	**50**	**62**	**80**	**100**

AT, assistive technologies; PwVI, persons with visual impairment.

The above finding was corroborated by the result of the qualitative strand. For instance, six PwVI (four from CoE and two from CoJ) indicated that ATs were very useful while four (one from CoE and three from CoJ) indicated that the devices and/or technologies were not useful.

### Difficulties faced by persons with visual impairment in using assistive technologies in Ekurhuleni and Johannesburg public libraries

The third objective of the study sought to establish the difficulties that PwVI faced in using ATs in CoE and CoJ libraries. This was necessary to recommend strategies for the alleviation of such difficulties for improved service delivery to PwVI. The realisation that several issues were likely to hinder ATs usage in those libraries, which could result in their nonusage, led to the focus on this aspect. The quantitative finding revealed the difficulty of inadequate or lack thereof of preferred ATs in libraries (CoE and CoJ), PwVI’s lack of ATs usage skills (CoE and CoJ), inadequate or lack of funding in libraries (CoE and CoJ), attitudes of staff (CoE) and lack of ATs’ knowledgeable personnel (CoJ). These are consistent with the below qualitative finding from the PwVI provided in verbatim responses:

‘The problem is that the facility I prefer to use is not available in the libraries visited.’ (55-year-old, male, CoE library user)

‘Some of us do not know how to use the devices, equipment or services due to lack of relevant usage skills.’ (62-year-old, male, CoJ library user)

‘Lack of proper marketing of the available devices resulting in users’ lack of awareness of their availability in libraries, and poor internet speed are some of the difficulties I experience during usage.’ (46-year-old, female, CoE library user)

‘Lack of staff support is a serious problem, especially for some of us who do not know how to use the devices or service.’ (56-year-old, male, CoJ library user)

A similar study by the LOC (2012) in the United States found that the major difficulty faced in providing library services to PwVI through NLS was PwVI’s (and their next of kin) lack of knowledge to operate the technology due to skills deficit. In South Africa, the National Library of South Africa (2014) identified limited financial and human resources as some of the difficulties faced by PwVI using ATs.

### Strategies for the alleviation of difficulties faced by persons with visual impairment in using assistive technologies at public libraries in Ekurhuleni and Johannesburg

Both respondents were further asked to provide their views on how the difficulties faced by the PwVI in using ATs could be alleviated for better future service planning by authorities. This question yielded several responses from librarians and PwVI. Notable, though, were the similarities in the given responses. For instance, both librarians and PwVI identified proper marketing of available ATs in public libraries; training of both PwVI and librarians on how to use and render services, respectively, using the available technologies; acquisition of additional but latest ATs devices and services; and establishing a partnership with other relevant stakeholders in the service providers, as ways to mitigate against the difficulties faced. The following are some of the responses provided by some librarians and PwVI users in this regard:

‘Proper marketing of the available ATs should be prioritised to increase user’s awareness of the available technological devices.’ (Librarian 1, female, CoE)

‘Staff members should be trained on how to interact and render services using the available assistive technologies. Furthermore, an adequate budget should be allocated to facilitate procurement of ATs as and when necessary.’ (Librarian 2, male, CoJ)

‘Latest computer technology should be procured so that PwVI can be able to use the library independently, and space should be reserved for persons with visual impairments in public libraries.’ (55-year-old, male, CoE library user)

‘Public libraries should partner with libraries for the blind so that they can benefit from their well-established stock consisting of a range of AT devices for the benefit of their PwVI.’ (56-year-old, male, CoJ library user)

‘Persons with visual impairment and organisations that represent them should be consulted when decisions are made on the types of ATs to be procured and service to be rendered as they know what is best for them. Further, properly skilled personnel should be appointed to render the AT-bases service.’ (46-year-old, female, CoE library user)

## Discussions

This study presents an overview of the availability and use of ATs for persons with a visual impairment at public libraries in the CoE and CoJ in South Africa. The outcome of this study can be used to guide the municipalities and policymakers to improve the current situation of inadequate availability and use of ATs as well as limitations for PwVI’s full participation in public libraries’ activities and services in the study settings. Assistive technology can play an important role towards realising the SDGs and UNCRPD goals that were ratified by South Africa in 2007, which could be achieved through, among others, the inclusion of PwVI in mainstream public libraries services. The findings raise concern about inadequate availability and use of ATs by persons with visual impairment, and serious issues hindering their availability and use include, but are not limited to, inadequate or the lack thereof of ATs procurement budget, staff attitudes towards ATs and PwVI, PwVI’s lack of skills to use ATs and the lack of proper marketing of AT-based services.

Given the above findings, authors in this study suggest that more still needs to be done in the face of inadequate availability of ATs for persons with a visual impairment. In fact, the potential action suggested by the authors following this finding is that an adequate budget should be made available to facilitate the procurement of the latest ATs that would meet the needs of users. It is common knowledge that there is inadequate, or lack thereof, services and resources meant for persons with visual impairment globally (Adetoro 2014; WHO 2007). Part of the reason for this is the lack of commitment by governments to support the service. The LIS sector in South Africa, for instance, had had trouble getting sufficient funds from the private sector and government (National Library of South Africa 2018). Metelerkamp and Banda (2022), for instance, reported that over the past few years, public libraries in both the Western Cape and Gauteng have been subjected to a steady decrease in budget allocations with libraries expected to do more with less. Inadequate availability of ATs is thus against the principles of fairness and equality of access to services for all as advocated by the IFLA Manifesto for Libraries Serving Persons with a Print Disability (2012). Public libraries in the two settings are, therefore, called upon to level the playing field for everyone to benefit in the information stream. The fact that ATs give hope to PwVI and other marginalised communities by enabling access to and use of library service implies that public libraries in Ekurhuleni and Johannesburg should invest more money in the AT-based service for the above call by IFLA to be realised. Hence, the availability of ATs such as computers with internet, CCTV and reading machines in public libraries should not be an option but a mandate if the government’s goal of ensuring equalisation of access to services for all is to be realised.

Against the finding that indicates that the usage of ATs by persons with visual impairment was inadequate, which could be attributed to reasons such as PwVI’s lack of AT usage skills, attitudes of staff towards ATs and PwVI as well as PwVI’s lack of awareness of their availability, the authors suggest that measures should be put in place to improve the current status quo. This means that PwVI should be trained on how to use ATs available, proper marketing should be done by libraries to improve users’ awareness of the AT-based services available, and the Library and Information Association of South Africa (LIASSA) should initiate and fund programmes and workshops that would empower library staff members to effectively work with and render AT-based services to the users. Dickard (2002) in Mamafha et al. (2016) argued that for users to effectively use the technology available in libraries, they should not only know about their availability but also should possess the relevant skills required to use them.

This study reported that PwVI experienced some difficulties in using ATs at public libraries in the two study settings. Notable were the similarities in most of the difficulties faced, which could be understood within the context of public libraries in the two cities sharing similar goals, services, values and system. The National Library of South Africa (2014) has identified problems such as financial and human resources as issues affecting the use of ATs in South Africa. The implication for this study is that all issues hindering service delivery to PwVI need to be addressed and opportunities created by them are exploited for public libraries to realise their goal of eliminating the exclusion of PwVI from accessing AT-based resources and services.

## Conclusion

The study concludes that certain types of ATs were inadequately available and used by PwVI at public libraries in the CoE and CoJ. This was, in part, due to a range of difficulties that require management attention if libraries are to make a difference in the lives of the people with a visual impairment. Given that access is a key factor in disability studies, future studies could investigate the extent to which ATs are accessible to persons with visual impairment in public libraries.
